# Transcultural validation of the “regulation of emotions questionnaire” among Tunisian university students

**DOI:** 10.1192/j.eurpsy.2021.1811

**Published:** 2021-08-13

**Authors:** E. Bouguira, U. Ouali, M. Ben Alaya, Y. Zgueb, F. Nacef

**Affiliations:** Department Psychiatry A, Razi Hospital, Manouba, Tunisia

**Keywords:** emotion regulation, validation, questionnaire, Depression

## Abstract

**Introduction:**

The regulation of emotions is a psychological process covering the capacity of inhibiting, maintaining or modulating emotions to create a coherent psychological functioning and to enable the individual to live in adequacy with his or her environment.

**Objectives:**

The aim of the study was to develop an Arabic version of the “Regulation of Emotions Questionnaire-2” (REQ-2T) from Phillips & Power, 2007, and to validate it in a sample of Tunisian university students.

**Methods:**

This is a validation study conducted in a sample of 384 Tunisian university students to whom we administered the REQ-2T, the DASS-21 questionnaire and a sociodemographic questionnaire. We tested face and content validity, reliability, and construct validity of the Arabic version of the REQ-2.

**Results:**

Face and content validity were satisfying. The internal consistency was average, with Cronbach’s alpha coefficient ranging from 0.44 to 0.65. The inter-dimensional correlation reflected statistically significant and logical correlations within the REQ-2T. Temporal stability was satisfying with a variable concordance of 0.65. Exploratory analysis revealed four factors similarly to the original version of the questionnaire. Statistically significant correlations were found between the REQ-2T and its external validator (DASS-21).

**Conclusions:**

The REQ-2T demonstrated good psychometric properties, thus this scale can be reliably used as a measure of emotion regulation in the Tunisian population.

**Disclosure:**

No significant relationships.

